# Supplementary material for: Orchestration of microtubules and the actin cytoskeleton in trichome cell shape determination by a plant-unique kinesin
Source: eLife. 2015 Aug 19;4:e09351. doi: 10.7554/eLife.09351 (PMC4574192; doi:10.7554/eLife.09351)
Supplement: Supplementary file 1. — List of primer sequences used in this study. DOI: http://dx.doi.org/10.7554/eLife.09351.027 [file elife09351s002.docx]

**Supplementary file 1. List of primer sequences used in this study.**

| Primer name | Primer sequences | Note |
| --- | --- | --- |
| KCBPP-F | CACCGTTTTGCAAGCTTTTTCCAT | CACC is for directional cloning. |
| GA-KCBP-R | **TGCGCCTGCGCCA**CTATCTGCCTCATCTTTTCGTG | The 12 bp fragment, bolded, is the reverse complement sequence of a “Glycine·Alanine” duplicates linker between KCBP and the FLAG tag to facilitate functional fusion. |
| KCBPP-R | CCACCCCGGTGAACAGCTCCTCGCCCTTGCTCACCATACCCGAAGAAAAAGAGAGATT | The 37 bp fragment underlined is the reverse complement sequence of 1-37 bp GFP-coding sequence to provide an overlap for subsequent fusion PCR. |
| PGFP-F | GTTCATCAGATCTACTACAATCTCTCTTTTTCTTCGGGTATGGTGAGCAAGGGCGAGGA | The 39 bp fragment underlined is the last 39 bp of the promoter. |
| GFP-R | AGCTCAACGAAGAATTACTGCCTCGTTGGCCCTCCAT**TGCGCCTGCGCC**CTTGTACAGCTCGTCCATG | The 37 bp fragment underlined is the reverse complement sequence of 1-37 bp *KCBP* genomic sequence; the 12 bp fragment bolded is the reverse complement sequence of a “Glycine·Alanine” duplicates linker. |
| KCBP1X-F | CGCCGGGATCACTCACGGCATGGACGAGCTGTACAAG**GGCGCAGGCGCA**ATGGAGGGCCAACGAGGCAG | The 37 bp fragment underlined is the last 37 bp of GFP; the 12 bp fragment bolded encodes a “Glycine·Alanine” duplicates linker. |
| KCBP1X-R | ATTTTACCATGCCATCACTT |  |
| KCBP2X-F | CACCCCGTTCAATGGTAGGAAT | CACC is for directional cloning. |
| KCBPP-∆NT-R | GGGGAAAGAAAAACCTTTTG**CAT**ACCCGAAGAAAAAGAGAGATTGTAGTAGA | The 23 bp fragment underlined is the reverse complement sequence of ATG (bolded) initiation codon and 454-473 bp of the *KCBP* genomic sequence. |
| KCBP-∆NT-F | ATCTCTCTTTTTCTTCGGGT**ATG**CAAAAGGTTTTTCTTTCCCCCCCTTTCTTC | The 23 bp fragment underlined is the last 20 bp of the promoter and ATG (bolded), the initiation codon. |
| KCBPP-∆MyTH4-R | AGCTTTCTACCGGTCAAAAGGGCTTCAATAAATGTGAAGCGCTCGCGG | The 29 bp fragment underlined is the reverse complement sequence of 992-1020 bp of the *KCBP* genomic sequence. |
| KCBP-∆MyTH4-F | CTGGCTCCAATGTCCGCGAGCGCTTCACATTTATTGAAGCCCTTTTGACCG | The 32 bp fragment underlined is 404-435 bp of the *KCBP* genomic sequence. |
| KCBPP-∆NT-MyTH4-R | AGCTTTCTACCGGTCAAAAGGGCTTCAAT**CAT**ACCCGAAGAAAAAGAGAGAT | The 32 bp fragment underlined is the reverse complement sequence of ATG (bolded) initiation codon and 454-473 bp of the *KCBP* genomic sequence. |
| KCBP-∆NT-MyTH4-F | ATCTACTACAATCTCTCTTTTTCTTCGGGT**ATG**ATTGAAGCCCTTTTGACCG | The 33 bp fragment underlined is the last 30 bp of the promoter and ATG (bolded) initiation codon. |
| KCBPP-∆FERM-R | CTAAAAAAAACTGAATTCCCGTATGGCAGTTCTTCACGACCAGGTGTGG | The 29 bp fragment underlined is the reverse complement sequence of 2135-2163 bp of the *KCBP* genomic sequence. |
| KCBP-∆FERM-F | CCTAGGCACACCACACCTGGTCGTGAAGAACTGCCATACGGGAATTCAGT | The 30 bp fragment underlined is 962-991 bp of the *KCBP* genomic sequence. |
| KCBPP-∆MyTH4-FERM-R | CTTGCGTACGCTAAAGCAAAGCATATCCTCAAATGTGAAGCGCTC | The 15 bp fragment underlined is the reverse complement sequence of 2159-2173 bp of the *KCBP* genomic sequence. |
| KCBP-∆MyTH4-FERM-F | GAGGATATGCTTTGCTTTAGCGTACGCAAGATAGATGATCCGATC | The 15 bp fragment underlined is 436-450 bp of the *KCBP* genomic sequence. |
| KCBPP-∆NT-MyTH4-FERM-R | CCGATCGGATCATCTATCTTGCGTACGCTAAA**CAT**ACCCGAAGAAAAAGAGAGATT | The 35 bp fragment underlined is the reverse complement sequence of 2159-2190 bp of the *KCBP* genomic sequence and ATG (bolded) initiation codon. |
| KCBP-∆NT-MyTH4-FERM-F | GATCTACTACAATCTCTCTTTTTCTTCGGGT**ATG**TTTAGCGTACGCAAGATAGATG | The 34 bp fragment underlined is the last 31 bp of the promoter and ATG (bolded) initiation codon. |
| KCBP-∆CC-Motor-CBD-R | **TGCGCCTGCGCC**AACCCCACGTTTGTTGATAC | The 12 bp fragment, bolded, is the reverse complement sequence of a “Glycine·Alanine” duplicates linker. |
| KCBP-∆CBD-R | **TGCGCCTGCGCC**TATATGTTTGCTGGGATCATTCAC | The 12 bp fragment, bolded, is the reverse complement sequence of a “Glycine·Alanine” duplicates linker. |
| KCBP-T982N-F | CAAACTGGTTCTGGAAAAAacTTCACTATATATG | The a, c in lower case indicate two mutated bases, the resulting AAC encodes the Asparagine (N). |
| KCBP-T982N-R | CATATATAGTGAAgtTTTTTCCAGAACCAGTTTG | The reverse complement sequence of the primer KCBP-T982N-F. |
| GFP-F | CACCATGGTGAGCAAGGGCGAGGAGCTG | CACC underlined is for directional cloning. |
| Sal I-GFP-F | CAATGTCGACAAATGGTGAGCAAGGGCGA | The 6 bp fragment underlined is *SaI* I enzyme recognition sequence. CAAT is protective bases of *Sal* I. |
| Not I-GFP-F | CTACGAGCGGCCGCCTTGTACAGCTCGTCCATGC | The 8 bp fragment, underlined, is *Not* I enzyme recognition sequence. CTACGA is protective bases of *Not* I. |
| Not I-CC-R | CTAAGCGGCCGCTGCGACATGCAATTCCTTA | The 8 bp fragment, underlined, is *Not* I enzyme recognition sequence. CTAA is protective bases of *Not* I. |
| NotI-FERM-R | CTAAGCGGCCGCCGGCTTAGAACTACAAGA | The 8 bp fragment, underlined, is *Not* I enzyme recognition sequence. CTAA is protective bases of *Not* I. |
| Not I-MyTH4-R | CTAAGAGCGGCCGCGGTCAAAAGGGCTTCAAT | The 8 bp fragment, underlined, is *Not* I enzyme recognition sequence. CTAAGA is protective bases of *Not* I. |
| Not I-NT-R | CTACGAGCGGCCGCAAAGCAAAGCATATCCTC | The 8 bp fragment, underlined, is *Not* I enzyme recognition sequence. CTACGA is protective bases of *Not* I. |
| GFP-MyTH4-R | GTGGGGATTGGATCCTTTTG**TGCTCCTGCTCC**CTTGTACAGCTCGTCCATGC | The 20 bp fragment underlined is the reverse complement sequence of 346-365 bp KCBP encoding sequence. The 12 bp fragment bolded is the reverse complement sequence of a “Glycine·Alanine” duplicates linker. |
| GFP-MyTH4-F | GCATGGACGAGCTGTACAAG**GGAGCAGGAGCA**CAAAAGGATCCAATCCCCAC | The 20 bp fragment underlined is the last 20 bp of GFP. The 12 bp fragment bolded encodes a “Glycine·Alanine” duplicates linker. |
| GFP-FERM-R | ATGGTTGTGAGCTTTCTACCCAT**TGCTCCTGCTCC**CTTGTACAGCTCGTCCATGC | The 20 bp fragment underlined is the reverse complement sequence of 826-845 bp of KCBP encoding sequence. The 12 bp fragment bolded is the reverse complement sequence of a “Glycine·Alanine” duplicates linker. |
| GFP-FERM-F | GCATGGACGAGCTGTACAAG**GGAGCAGGAGCA**ATGGGTAGAAAGCTCACAACCA | The 20 bp fragment underlined is the last 20 bp of GFP. The 12 bp fragment bolded encodes a “Glycine·Alanine” duplicates linker. |
| TUB6-P-F | CACCTTTGGATATAAACACAAAAACCAATGATACGAACAGATGC | CACC is for directional cloning. |
| mCherry-TUB6-P-R | GCCCTTGCTCACCATCTTCTATTTTATCTGAAATCAACATTACATGAACAAAACAG | The 15 bp fragment underlined is the reverse complement sequence of the first 15 bp of mCherry encoding sequence. |
| TUB6-P-mCherry-F | CAGATAAAATAGAAGATGGTGAGCAAGGGCGAGGATAAC | The 18 bp fragment underlined is the last 18bp of the promoter of AtTUB6. |
| TUB6-GA-mCherry-R | AAGGATTTCTCTCAT**TGCGCCTGCGCC**CTTGTACAGCTCGTCCATGCCGCC | The 15 bp fragment underlined is the reverse complement sequence of the first 15 bp of AtTUB6 encoding sequence. The 12 bp fragment bolded is the reverse complement sequence of a “Glycine·Alanine” duplicates linker. |
| mCherry-GA-12250-F | GACGAGCTGTACAAG**GGCGCAGGCGCA**ATGAGAGAAATCCTTCACATTCAAGGTGGTCA | The 15 bp fragment underlined is the last 15 bp of mCherry encoding sequence. The 12 bp fragment bolded encodes a “Glycine·Alanine” duplicates linker. |
| TUB6-R | **TGCGCCTGCGCC**CTCATGATCCAATATCTCTTCTTCATCC | The 12 bp fragment bolded is the reverse complement sequence of a “Glycine·Alanine” duplicates linker. |
| 031704-LP | CCTCCCAACAGGATAGTTTCC |  |
| 031704-RP | CATCTGATCGATCTACGCCTC |  |
| LBb1.3 | ATTTTGCCGATTTCGGAAC |  |
| 031704-F1 | GGGTATGGAGGGCCAACGAGGCAGTAATTC |  |
| 031704-R2 | GCAAAGCATATCCTCAAATGTGAAGCGC |  |
| 031704-F2 | TCCTCTGGCTCCAATGTC |  |
| 031704-R4 | CCGGGATCAGATGATTTA |  |
| UBQ5-F | GACGCTTCATCTCGTCC |  |
| UBQ5-R | CCACAGGTTGCGTTAG |  |
